# Supplementary material for: Structural insights into the functional mechanism of the ubiquitin ligase E6AP
Source: Nat Commun. 2024 Apr 26;15:3531. doi: 10.1038/s41467-024-47586-w (PMC11053172; doi:10.1038/s41467-024-47586-w)
Supplement: Supplementary file 1 — Supplementary Information [file 41467_2024_47586_MOESM1_ESM.pdf]

Supplementary Information

Structural insights into the functional mechanism of the ubiquitin ligase E6AP

Zhen Wang, Fengying Fan, Zhihai Li, Fei Ye, Qingxia Wang, Rongchao Gao, Jiaxuan Qiu, Yixin Lv, Min Lin, Wenwen Xu, Cheng Luo, Xuekui Yu

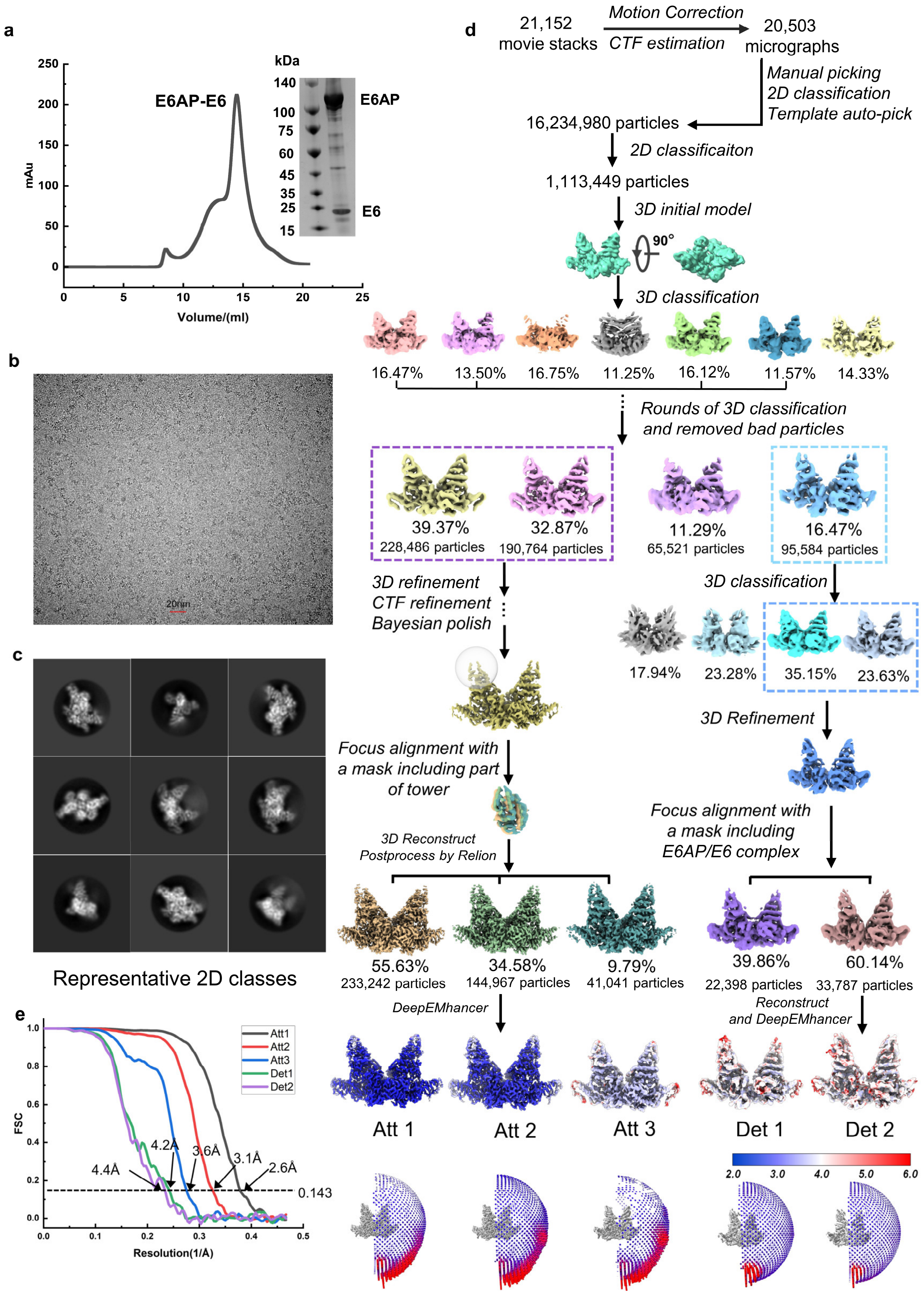

Supplementary Fig. 1 Sample preparation and image processing of the E6AP/E6 complex. a, Size-exclusion chromatography profile and SDS-PAGE analysis of the E6AP/E6 complex co-expressed in insect cells. **b,** Representative cryo-EM micrograph. **c,** Representative 2D classes. **d,** Flow chart of the image processing. The final 3D

reconstructions are colored according to local resolution. The corresponding angular distribution of particles used for the final reconstruction is depicted. **e,** Gold-standard Fourier shell correlation (FSC) curves of the final 3D reconstructions. Source data are provided as a Source Data file.

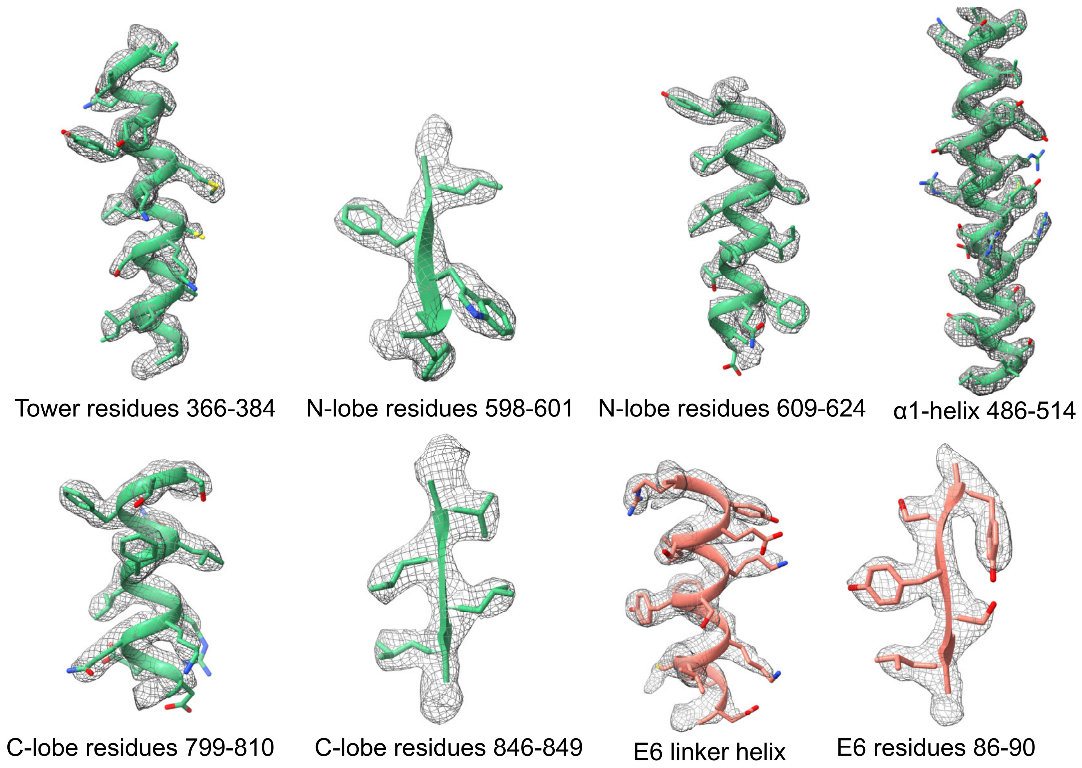

Supplementary Fig. 2 Representative density maps of the E6AP/E6 complex. The density maps and models are shown as gray meshes and ribbons, respectively. The side chains of residues are shown as sticks.

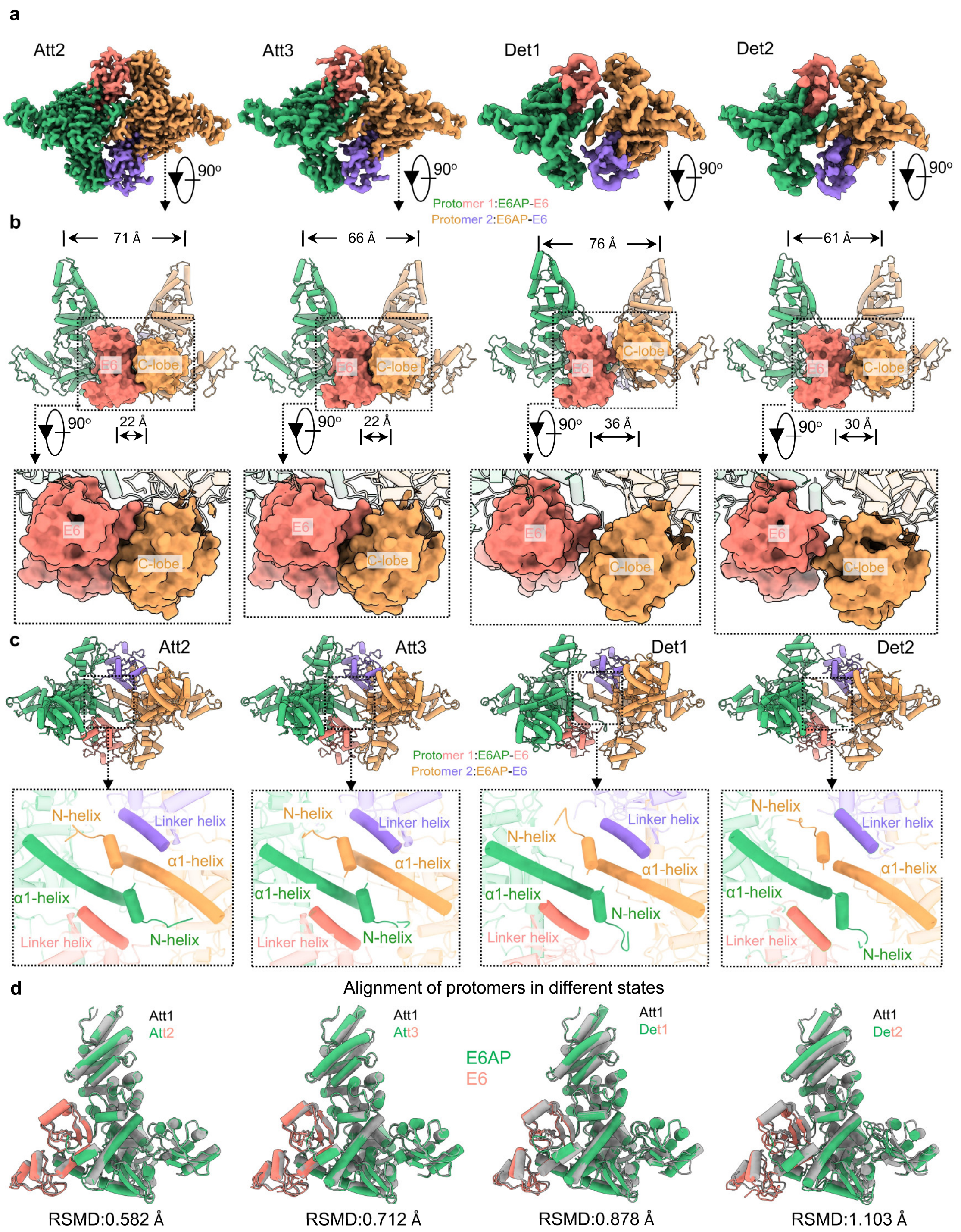

Supplementary Fig. 3 Density maps and models of the E6AP/E6 complex in Att2, Att3, Det1, and Det2 states. a, Bottom view showing the density maps of the E6AP/E6 complex in four different states. The E6AP/E6 complex in each state is a dimer of the E6AP/E6 protomer. E6AP and E6 are green and red, respectively, in protomer 1 and yellow and purple, respectively, in protomer 2. Maps are processed by DeepEMhancer. **b-c,** Side (**b**) and top (**c**) views showing the models of the E6AP/E6 complex in four

different states. In (**b**), the E6 in protomer 1 and the C-lobe in protomer 2 are shown in surface mode. The distances between two towers or between the E6 in protomer 1 and the C-lobe in protomer 2 are labeled. Close-up view of the regions outlined with black lines in (**b**) shows the spatial relationship between the E6 in protomer 1 and the C-lobe in protomer 2. E6 and the C-lobe are attached in Att2 and Att3 states while detached in Det1 and Det2 states. A close-up view of the regions outlined with black lines in (**c**) shows the two 3-helix bundles at the interface between two E6AP/E6 protomers. The N-helix and α1-helix from two protomers form the inter-protomer interface. **d,** Structural alignment of one E6AP/E6 protomer in Att1 with that in the other four states.

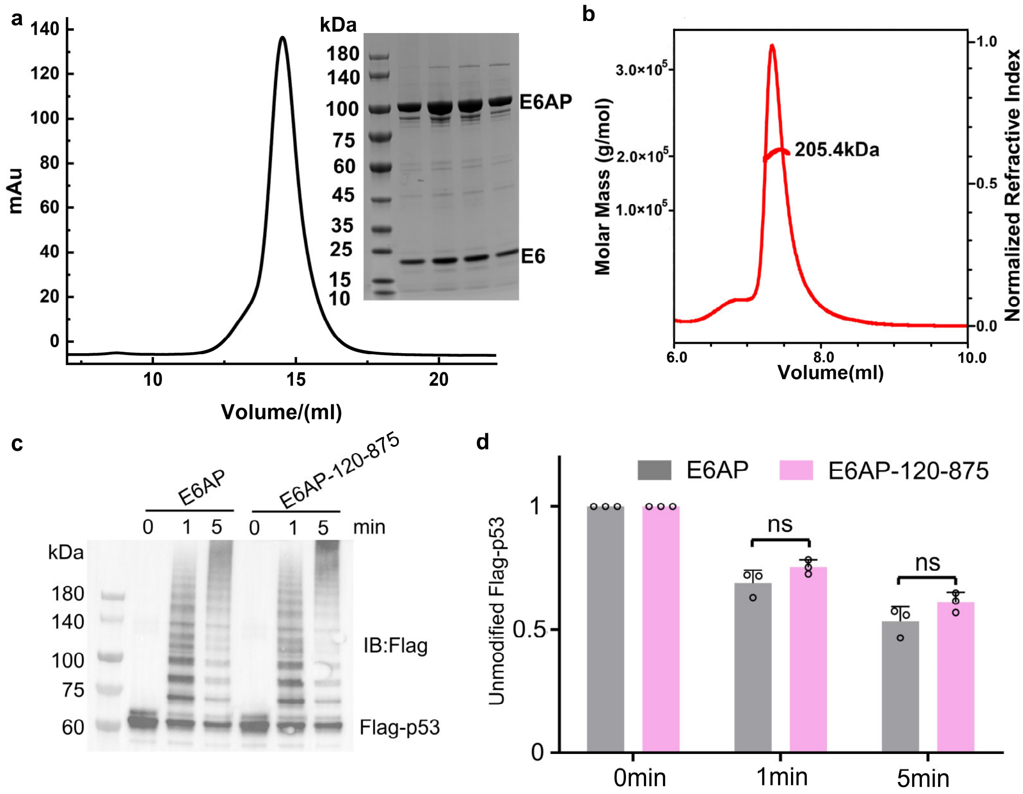

Supplementary Fig. 4 E6AP N-terminal truncation mutant (residues 120-875) forms a functional complex with E6. a, Size-exclusion chromatography (SEC) profile and SDS-PAGE analysis of the sample co-purified with the strep-tagged E6AP. The E6AP N-terminal truncation mutant and E6 were co-expressed in insect cells. The expressed sample was purified using Strep-tag affinity beads and the resultant sample analyzed using SEC. **b,** Static light-scattering measurements of the molecular weights of the purified complex in **(a)**. The theoretical molecular weight of the complex (dimer of the E6AP mutant/E6 protomer) is 211.2 kDa. **c,** Effect of E6AP N-terminal truncation on the ubiquitination of the substrate p53. The activity of E6AP toward p53 is negatively correlated with the amount of unmodified p53. **d**, Statistics of the enzymatic activity in **(c)**. Data are presented as the mean ± SD of triplicate experiments. ns, not significant, based on one-way analysis of variance (ANOVA) with Tukey’s multiple comparison test. Source data are provided as a Source Data file.

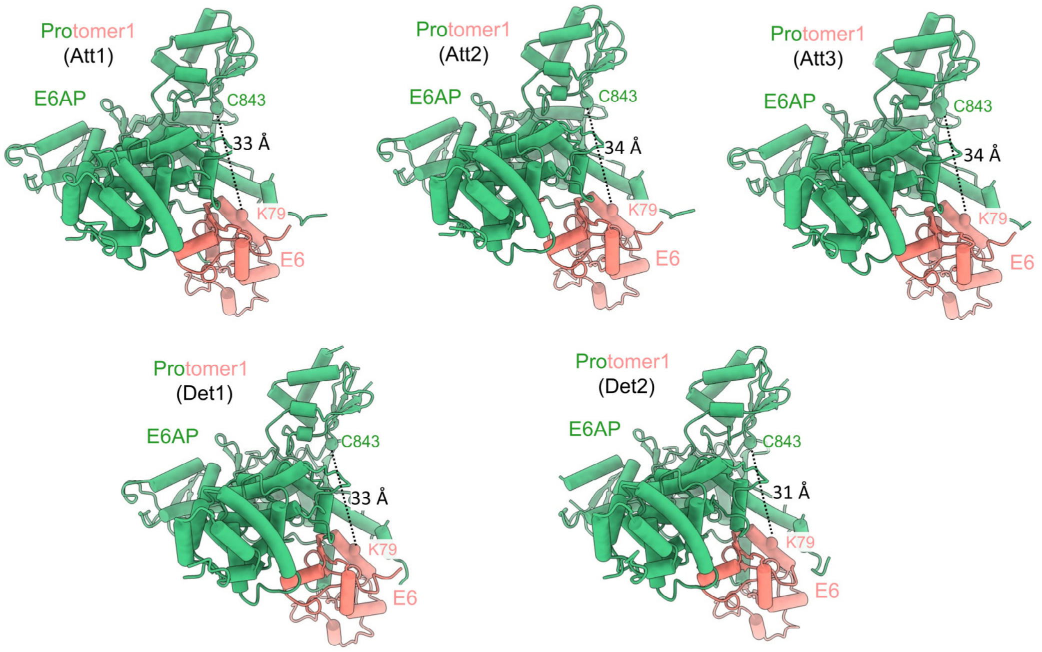

Supplementary Fig. 5 Distances between the catalytic C843 residue in the C-lobe and the lysine (K79) of E6 in one E6AP/E6 protomer. Within one E6AP/E6 protomer, among all lysine residues of E6, K79 is closest to C843 of E6AP. The spatial relationship between the two residues is shown using one protomer of the E6AP/E6 complex in five states. The two residues are displayed as balls. E6AP and E6 are green and red, respectively.

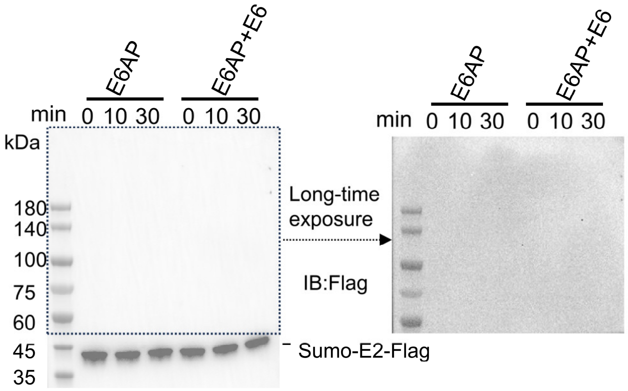

Supplementary Fig. 6 UbcH7 is not ubiquitinated by E6AP. The ubiquitination assay followed the same procedure as described in Figure 3f, with the only difference being the addition of a Flag tag at the C-terminus of Sumo-UbcH7 for detection by immunoblotting (IB). The experiment was repeated three times with similar results. Source data are provided as a Source Data file.

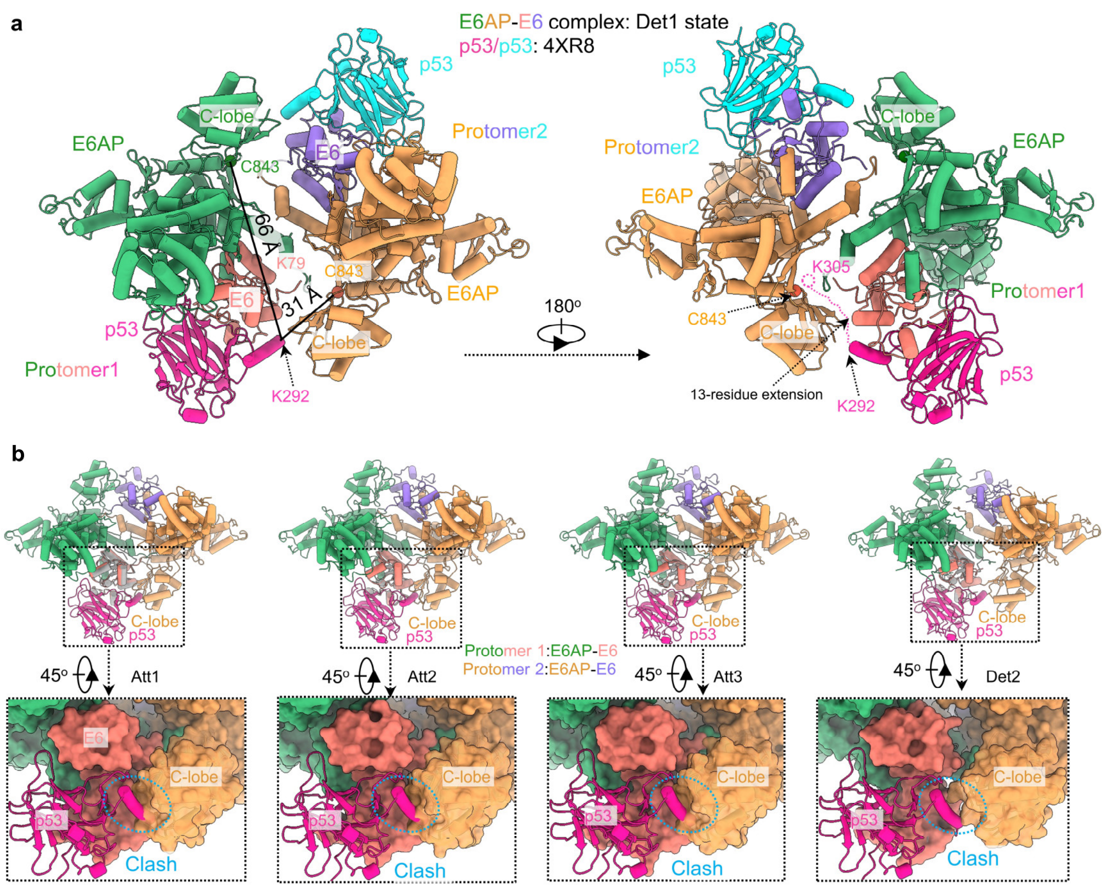

Supplementary Fig. 7 Structural alignment of the E6AP/E6 complex in each state with the E6AP peptide/E6/p53 core complex. a, Comparative model of the E6AP/E6/p53 core complex showing the spatial relationship between p53 and the C-lobe. The model is produced by superimposing the structure of the E6AP/E6 complex with the crystal structure of the E6AP peptide/E6/p53 core domain (PDB ID: 4XR8). In one protomer, the C843 residue of the C-lobe of E6AP, K79 of E6, and the C-terminal residue K292 of the p53 core are in one line. In the model on the right, a red hollow circle represents the major ubiquitination site (K305) of p53 by E6AP, whereas the red dotted line represents the unmodelled region (13 residues) of the p53 core from K292 to K305. **b,** Structural alignment of the E6AP/E6 complex in each of the four conformations (Att2, Att3, Det1 and Det2) with the E6AP peptide/E6/p53 core complex. Close-up views of the outlined regions shows that the p53 core would clash with the C-lobe. E6AP, E6 and p53 are green, red and dark red respectively in protomer 1 and yellow, purple and cyan respectively in protomer 2.

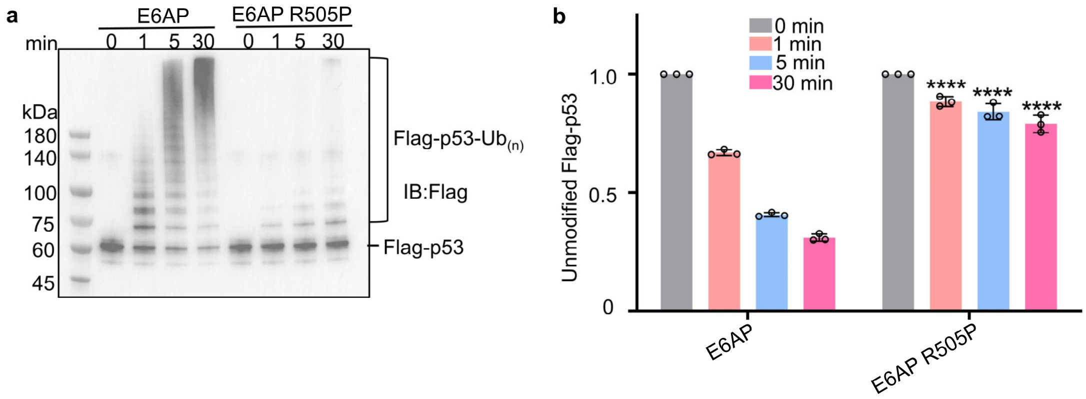

Supplementary Fig. 8 Effect of the E6AP R505P mutation on the ubiquitination of p53. a, Western blot assay for the ubiquitination of p53 by the E6AP R505P mutant. **b,** Statistics of the enzymatic activity in (**a**). Statistical significance tests compare the amount of unmodified p53 by the E6AP R505P mutant with that by wild-type E6AP at the same time point. Data are presented as the mean ± SD of triplicate experiments. ****$p < 0.0001$ based on one-way analysis of variance (ANOVA) with Tukey's multiple comparison test. Source data are provided as a Source Data file.

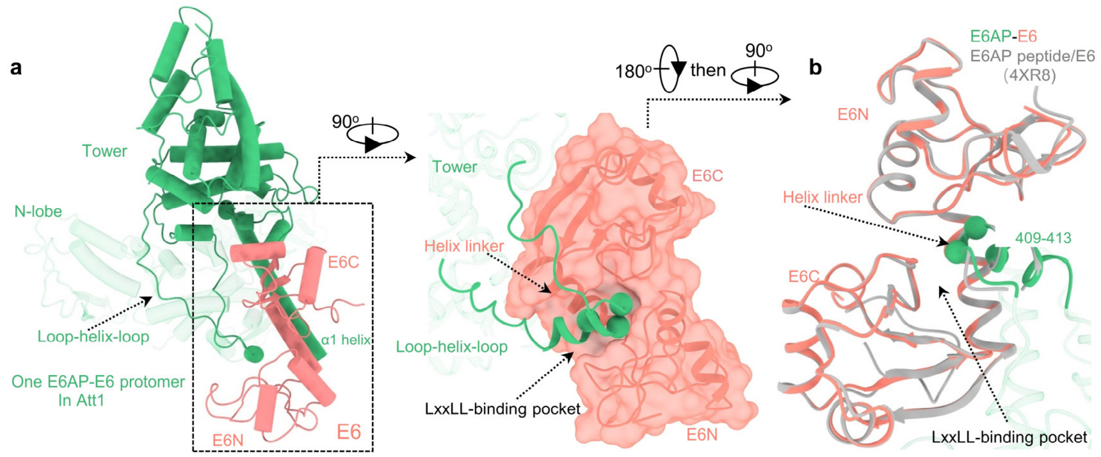

Supplementary Fig. 9 Interactions between E6 and the LxxLL motif of E6AP. a, The LxxLL motif from the loop-helix-loop element of E6AP (green) in Att1 inserts into a cleft formed by E6N and E6C of E6 (red). For clarity, only one protomer is shown. Leucine residues in the E6 binding motif (LxxLL) of E6AP are shown as balls. **b,** Structural comparison of the E6AP/E6 complex with the E6AP peptide/E6 (4XR8, grey) complex.

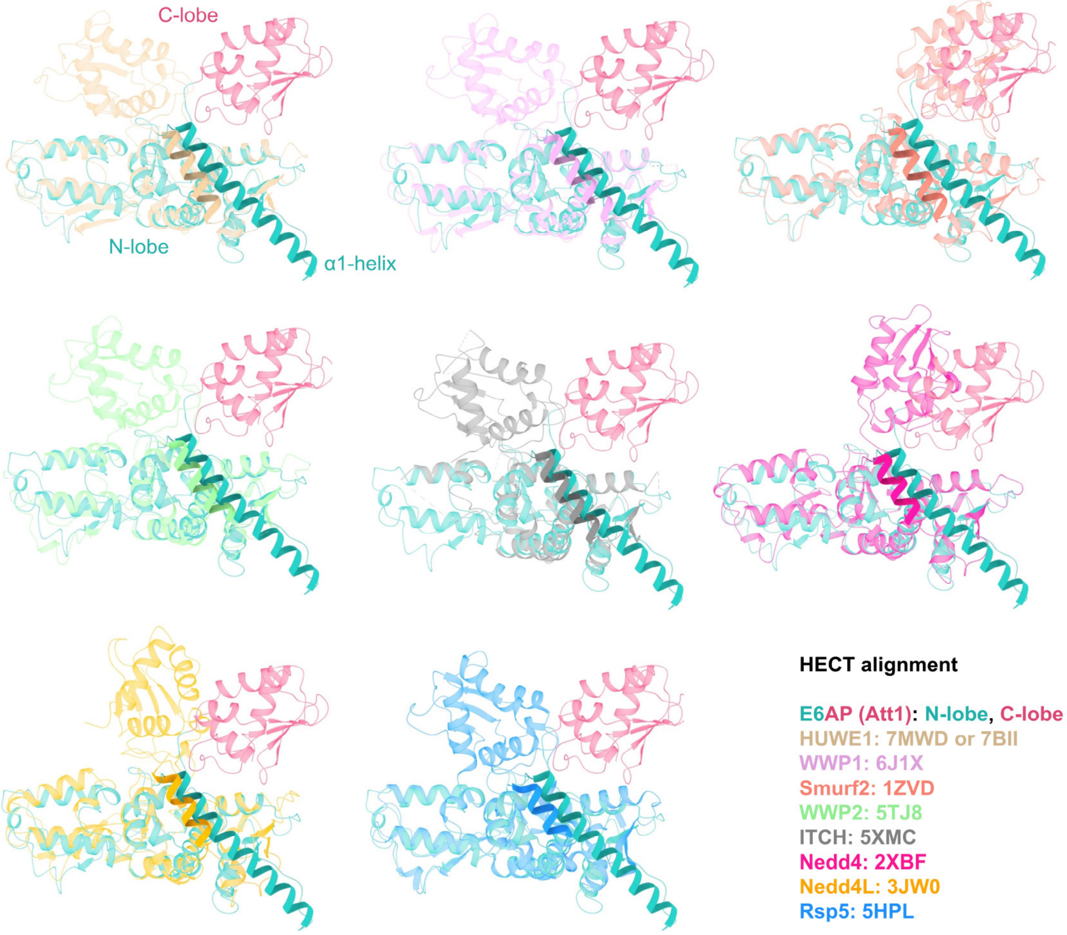

Supplementary Fig. 10 Superimposition of the E6AP HECT domain in the Att1 state with that of other known HECT-type ubiquitin ligases. 7MWD and 7BII are the entry IDs of structures of human HUWE1 and Nematocida HUWE1, respectively. The conformations of the α1-helices are almost identical in the two structures. Only one structure is shown for clarity. N-lobe and C-lobe of E6AP are green and red, respectively. The color codes for HECT of other known HECT-type ubiquitin ligases are indicated.

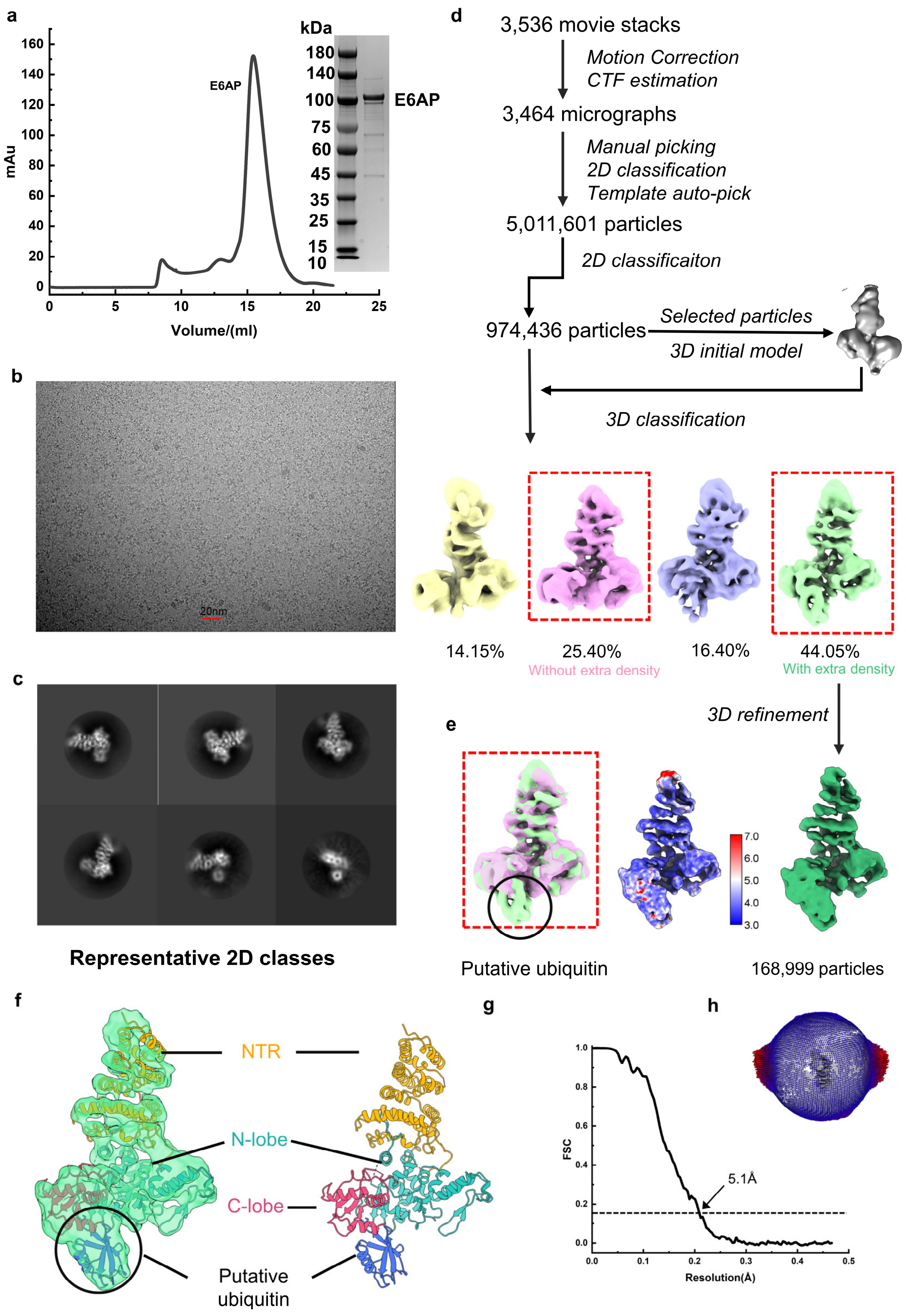

Supplementary Fig. 11 Sample preparation and image processing of E6AP alone. **a,** Size-exclusion chromatography profile and SDS-PAGE analysis of E6AP for cryo-EM grid preparation. **b,** A representative cryo-EM micrograph. **c,** Representative 2D classes. **d,** Flow chart of the image processing. **e,** Alignment of two maps reconstructed in (**d**). **f,** Density map and model of E6AP. Different parts of the E6AP model in Att1

are rigidly docked into the density map of the E6AP monomer. The extra density is located near the active site C843 of the C-lobe and fits well with known structures and representations of ubiquitin. **g,** Gold-standard Fourier shell correlation (FSC) curves of the final 3D reconstruction of the extra density in (**f**). **h,** A representation of the angular distribution of particles used in the final reconstruction. Source data are provided as a Source Data file.

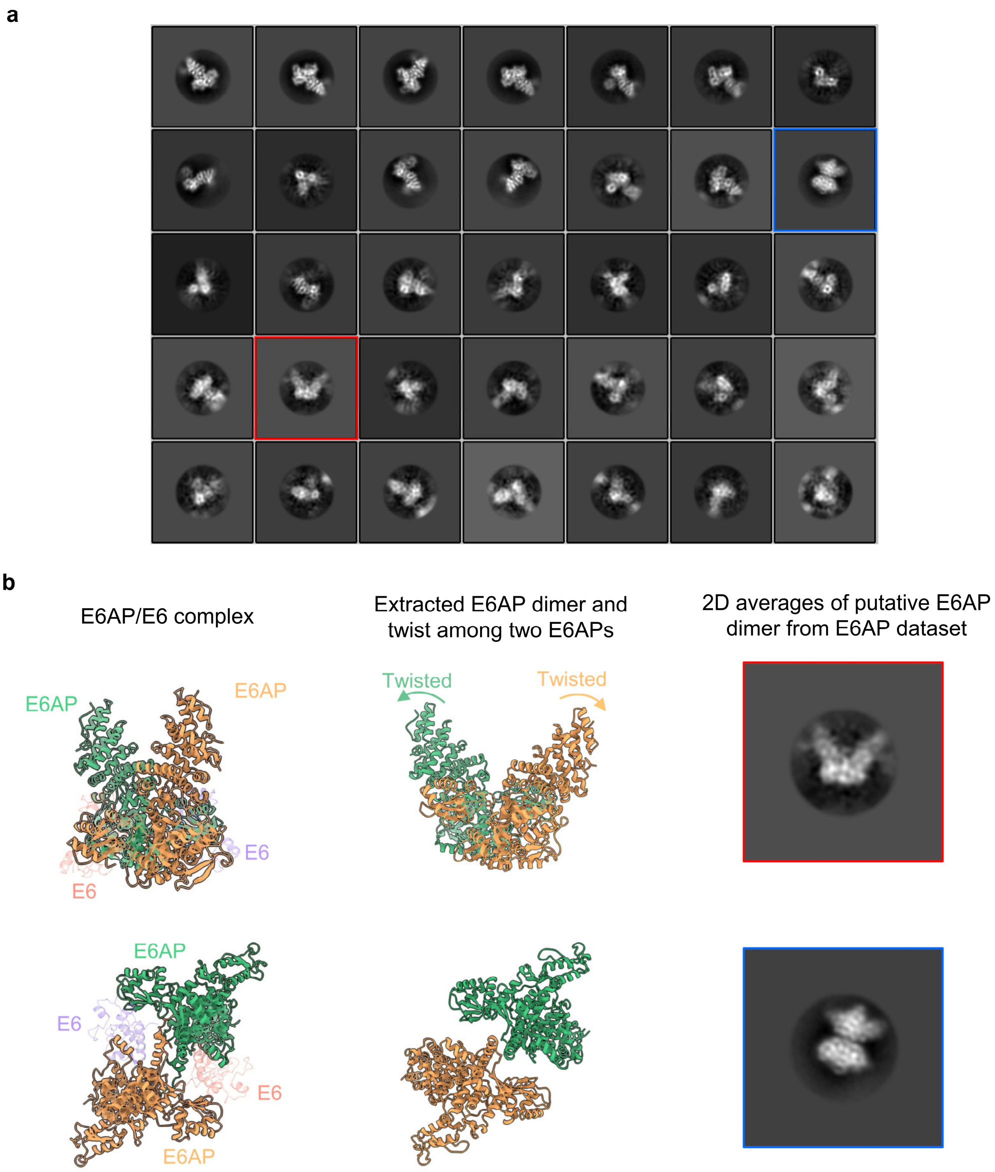

Supplementary Fig. 12 Putative E6AP dimer illustrated by the 2D averages of the E6AP dataset. a, A snapshot of an intermediate result of the 2D classification of the E6AP dataset. 2D views representing projections of the putative E6AP dimer are boxed in red and blue. **b**, Comparison of the selective 2D averages of the E6AP dataset with the E6AP/E6-derived E6AP dimer structure. The E6AP/E6 complex structure (left), an artificial model of the E6AP dimer (middle, extracted from the structure of the E6AP/E6 complex structure and twisted between the two E6AP molecules), and the boxed 2D averages in (**a**) of the E6AP dataset (right) are shown side by side. E6AP and E6 are green and red, respectively, in protomer 1 and yellow and purple, respectively, in protomer 2.

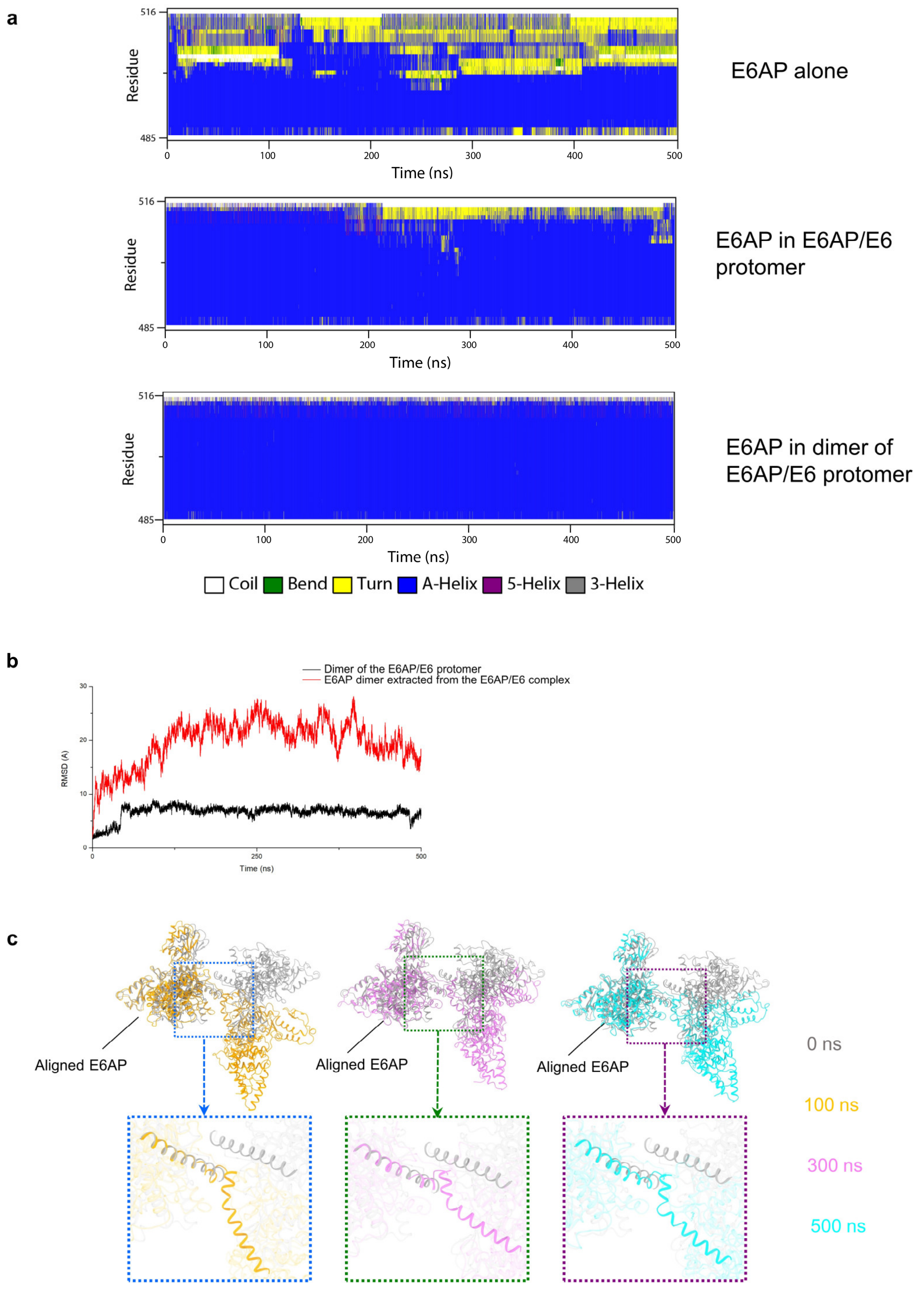

Supplementary Fig. 13 E6 binding stabilizes the extended α1-helix. a, Secondary structure evolution of the α1-helix (486-514) in trajectory for the dimer of the E6AP/E6 protomer, one E6AP/E6 protomer, and the isolated E6AP, as calculated using the DSSP program. The structures of the E6AP/E6 protomer and the isolated E6AP are extracted directly from the structure of the E6AP/E6 complex. **b,** Cα RMSD plots for the E6AP dimer over the 500-ns simulation for each of the two systems containing the dimer of

the E6AP/E6 protomer (black) and the E6AP dimer (as extracted from the E6AP/E6 complex, red). It shows that without E6, the E6AP dimer is much less stable. **c,** Alignment of the snapshot structures during the 500-ns simulation for the system containing the E6AP dimer. The initial structure and snapshot structures at 100, 300, and 500 ns are colored in gray, yellow, red, and blue respectively. The initial structure is extracted from the E6AP/E6 complex. The zoomed views below show the distorted interface between two E6AP molecules. The extended parts of the α1-helices are kinked or distorted. Source data are provided as a Source Data file.

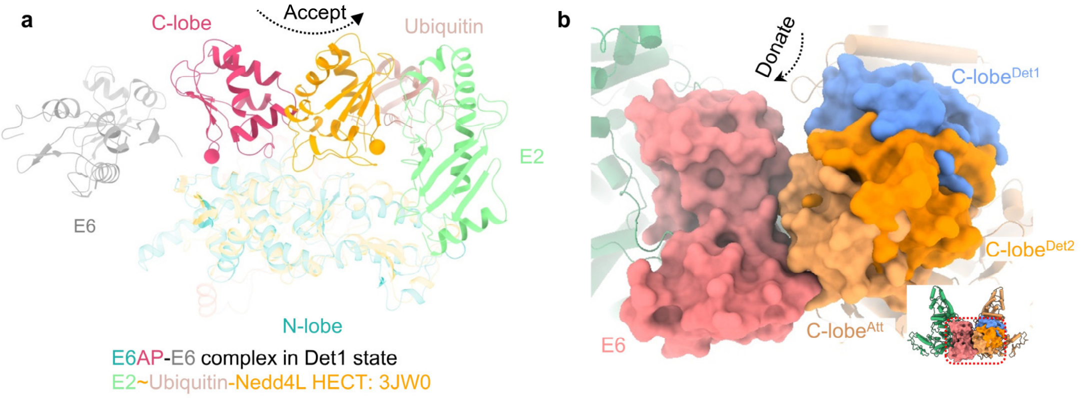

Supplementary Fig. 14 Ubiquitin transfer facilitated by dynamics of the E6AP/E6 complex. a, Structural alignment of the E6AP/E6 complex in Det1 and the E2 (light green)/ubiquitin (brown)/Nedd4L HECT (yellow) complex (PDB ID: 3JW0). N-lobe and C-lobe of E6AP are green and red, respectively. E6 is in grey. The C-lobe in both Det conformations (Det1 and Det2) is free to move and accept ubiquitin from the E2. The active site cysteines of the E3s are shown as balls, which can accept ubiquitin from the E2. For clarity, the alignment uses the Det1 state to represent both Det states. **b**, Structural alignment showing the spatial relationship between the E6 (red) of one protomer and the catalytic C-lobe of another protomer in Det1 (blue), Det2 (orange), and Att (yellow) states. Alignment was performed by aligning one E6AP/E6 protomer. For clarity, the alignment uses the Att1 state to represent all three Att states and hides the E6AP except for the C-lobe and E6 in Det states. The small cartoon in the lower right corner is shown for orientation purposes; a close-up view of the region is enclosed by a red line.

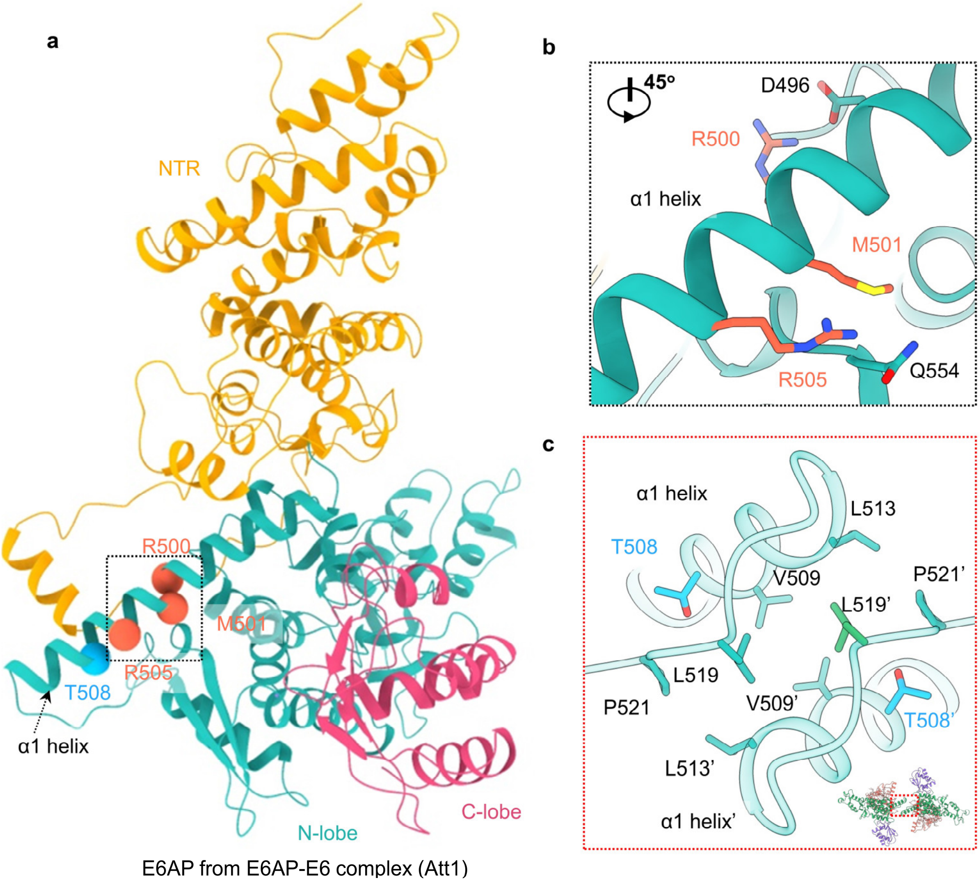

Supplementary Fig. 15 Effect of E6AP mutations which are associated with Angelman syndrome or Autism. a, Structural mapping of mutations on the extended α1-helix. N-terminal region (NTR), N-lobe and C-lobe of E6AP are yellow, green, and red, respectively. The Autism-related mutation site is labeled as a blue ball, while the Angelman syndrome-related mutation sites are labeled as orange balls. **b,** Detailed interactions of the Angelman syndrome-related mutation sites. **c**, Location of the Autism-related mutation site. The apostrophe symbol (') indicates cognate residues on the other protomer. T508 is located at the hydrophobic interface of two E6AP molecules.

Extended Data Table 1 Cryo-EM data collection, refinement, and validation statistics.

	E6AP/E6 complex					E6AP
	Att1	Att2	Att3	Det1	Det2	
**Data collection and processing**						
Magnification			81,000			81,000
Voltage(kV)			300			300
Electron exposure(e^{-}/Å^2)			50			70
Defocus range(㎛)			-1.5 to -2.0			-1.5 to -2.0
Pixel size(Å)			1.071			1.071
Symmetry imposed			C2			C1
Initial particle images(no.)			16,234,980			5,011,601
Final particle images(no.)	233,242	144,967	41,041	22,398	33,787	168,999
Map resolution(Å)	2.6	3.1	3.6	4.2	4.4	5.1
FSC threshold	0.143	0.143	0.143	0.143	0.143	0.143
**Refinement**						
Model resolution(Å)	2.7	3.1	3.7	4.2	6.4	--
FSC threshold	0.5	0.5	0.5	0.5	0.5	--
Model Composition						
Non-hydrogen atoms	13,812	13,812	13,770	13,388	12,456	--
Protein residues	1,688	1,688	1,688	1,656	1,582	--
Ligands (Zn)	4	4	4	4	4	--
B factors(Å^2)						
Protein	65.18	149.88	97.27	132.77	153.07	--
Ligand	1114.15	7282.68	23.48	33.59	38.08	--
R.m.s.deviations						
Bond length(Å^2)	0.004	0.003	0.003	0.007	0.008	--
Bond angle(°)	0.538	0.517	0.554	1.087	1.076	--
**Validation**						
Molprobity score	1.51	1.53	1.67	2.12	2.23	--
Clashscore	9.78	7.09	9.61	12.95	15.44	--
Ramachandran plot						
Favored(%)	98.33	97.25	97.07	91.77	90.28	--
Allowed(%)	1.67	2.75	2.93	8.23	9.72	--
Outliers(%)	0.00	0.00	0.00	0.00	0.00	--

Extended Data Table 2 Primers used in this study

Primer name	Primer sequences (5'-3')
E6AP-1-Forward	CACCCGCAGTTCGAAAAAATGGAGAAGCTGCACCAG
E6AP-120-Forward	CCGCAGTTCGAAAAAATTGATTTTAAAGATG
E6AP-875-Reverse	CTAGTACTTCTCGACAAGCTTTTACAGCATGCCAAATCCTTTG
E6AP-R505P-Forward	CGCATGTACAGTGAACCAAGAATCACTGTTCTC
E6AP-R505P-Reverse	GAGAACAGTGATTCTTGGTTCACTGTACATGCG
E6AP-C843A-Forward	CCTACATCTCATACTGCATTTAATGTGCTTTTAC
E6AP-C843A-Reverse	GTAAAAGCACATTAAATGCAGTATGAGATGTAGG
p53-1-Forward	GATGATAAGGGTTCAGCT ATGGAGGAGCCGCAGTCAGATC
p53-393-Reverse	GTACTTCTCGACAAGCTTTCAGTCTGAGTCAGGCC
E6-1-Forward	CCAAGGTGGTAGCAGCATGCACCAAAAGAGAACTGC
E6-158-Reverse	GTACTTCTCGACAAGCTTTTACAGCTGGGTTTC
E6-D32A-Forward	GCAGACCACCATCCACGCGATCATCCTGGAATGCG
E6-D32A-Reverse	CGCATTCCAGGATGATCGCGTGGATGGTGGTCTGC
E6-Y50A-Forward	CTGCGTCGTGAAGTTGCGGATTTCGCGTTCCGTG
E6-Y50A-Reverse	CACGGAACGCGAAATCCGCAACTTCACGACGCAG
E6-R148A-Forward	TGCATGAGCTGCAGCGCGAGCTCTCGTACCCGTC
E6-R148A-Reverse	GACGGGTACGAGAGCTCGCGCTGCAGCTCATGCA
E6-F76A/I80A/Y83A-Forward	GATAAATGCCTGAAAGCGTACAGCAAAGCGAGCGAAGCGCGTCACTACAGCTAC
E6-F76A/I80A/Y83A-Reverse	GTAGCTGTAGTGACGCGCTTCGCTCGCTTTGCTGTACGCTTTCAGGCATTTATC
E6-Y88A/Y91A-Forward	GAATACCGTCACTACAGCGCGTCTCTGGCGGGCACCACCCTGGAAC
E6-Y88A/Y91A-Reverse	GTTCCAGGGTGGTGCCCGCCAGAGACGCGCTGTAGTGACGGTATTC
