# Supplementary material for: Structural insights into the functional mechanism of the ubiquitin ligase E6AP
Source: Nat Commun. 2024 Apr 26;15:3531. doi: 10.1038/s41467-024-47586-w (PMC11053172; doi:10.1038/s41467-024-47586-w)
Supplement: Supplementary file 2 — Reporting Summary [file 41467_2024_47586_MOESM2_ESM.pdf]

nature portfolio

Corresponding author(s): Xuekui Yu

Last updated by author(s): 2024-03-23

Reporting Summary

Nature Portfolio wishes to improve the reproducibility of the work that we publish. This form provides structure for consistency and transparency in reporting. For further information on Nature Portfolio policies, see our Editorial Policies and the Editorial Policy Checklist.

Statistics

For all statistical analyses, confirm that the following items are present in the figure legend, table legend, main text, or Methods section.

n/a	Confirmed	
☐	☒	The exact sample size (*n*) for each experimental group/condition, given as a discrete number and unit of measurement
☐	☒	A statement on whether measurements were taken from distinct samples or whether the same sample was measured repeatedly
☐	☒	The statistical test(s) used AND whether they are one- or two-sided<br>*Only common tests should be described solely by name; describe more complex techniques in the Methods section.*
☒	☐	A description of all covariates tested
☒	☐	A description of any assumptions or corrections, such as tests of normality and adjustment for multiple comparisons
☐	☒	A full description of the statistical parameters including central tendency (e.g. means) or other basic estimates (e.g. regression coefficient) AND variation (e.g. standard deviation) or associated estimates of uncertainty (e.g. confidence intervals)
☐	☒	For null hypothesis testing, the test statistic (e.g. *F*, *t*, *r*) with confidence intervals, effect sizes, degrees of freedom and *P* value noted<br>*Give P values as exact values whenever suitable.*
☒	☐	For Bayesian analysis, information on the choice of priors and Markov chain Monte Carlo settings
☒	☐	For hierarchical and complex designs, identification of the appropriate level for tests and full reporting of outcomes
☒	☐	Estimates of effect sizes (e.g. Cohen's *d*, Pearson's *r*), indicating how they were calculated

Our web collection on statistics for biologists contains articles on many of the points above.

Software and code

Policy information about availability of computer code

Data collection	SerialEM v3.7
Data analysis	Relion v3.0, MotionCor2 v1.2.2, Gctf 1.06, PHENIX-1.14, COOT 0.8.9.1, Chimera 1.17.3, ChimeraX v1.3, ResMap v1.1.4, ASTRA 6, Maestro version 9.0, PROPKA, Gromacs 2019.6, Prism 6.

For manuscripts utilizing custom algorithms or software that are central to the research but not yet described in published literature, software must be made available to editors and reviewers. We strongly encourage code deposition in a community repository (e.g. GitHub). See the Nature Portfolio guidelines for submitting code & software for further information.

Data

Policy information about availability of data

All manuscripts must include a data availability statement. This statement should provide the following information, where applicable:

- Accession codes, unique identifiers, or web links for publicly available datasets
- A description of any restrictions on data availability
- For clinical datasets or third party data, please ensure that the statement adheres to our policy

All density maps generated in this study have been deposited in the Electron Microscopy Bank under accession codes EMD-36604 [https://www.ebi.ac.uk/pdbe/entry/emdb/EMD-36604] (the monomeric E6AP with a putative ubiquitin), EMD-36599 [https://www.ebi.ac.uk/pdbe/entry/emdb/EMD-36599] (the E6AP/E6 complex in the Att1), EMD-36600 [https://www.ebi.ac.uk/pdbe/entry/emdb/EMD-36600] (the E6AP/E6 complex in the Att2), EMD-36601 [https://www.ebi.ac.uk/pdbe/entry/emdb/EMD-36601] (the E6AP/E6 complex in the Att3), EMD-36602 [https://www.ebi.ac.uk/pdbe/entry/emdb/EMD-36602] (the E6AP/E6 complex in the [illegible]

Det1), and EMD-36603 [https://www.ebi.ac.uk/pdbe/entry/emdb/EMD-36603] (the E6AP/E6 complex in the Det2). The atomic coordinates generated in this study have been deposited in the Protein Data Bank under accession code 8JRN [https://doi.org/10.2210/pdb8jrn/pdb] (the E6AP/E6 complex in the Att1), 8JRO [https://doi.org/10.2210/pdb8jro/pdb] (the E6AP/E6 complex in the Att2), 8JRP [https://doi.org/10.2210/pdb8jrp/pdb] (the E6AP/E6 complex in the Att3), 8JRQ [https://doi.org/10.2210/pdb8jrq/pdb] (the E6AP/E6 complex in the Det1), and 8JRR [https://doi.org/10.2210/pdb8jrr/pdb] (the E6AP/E6 complex in the Det2). Source data are provided with this paper. The previously-published atomic coordinates used in this study have been deposited in the Protein Data Bank under accession code 7MWD [https://doi.org/10.2210/pdb7mwd/pdb], 7BII [https://doi.org/10.2210/pdb7bii/pdb], 6J1X [https://doi.org/10.2210/pdb6j1x/pdb], 1ZVD [https://doi.org/10.2210/pdb1zvd/pdb], 5TJ8 [https://doi.org/10.2210/pdb5tj8/pdb], 5XMC [https://doi.org/10.2210/pdb5xmc/pdb], 2XBF [https://doi.org/10.2210/pdb2xbf/pdb], 3JW0 [https://doi.org/10.2210/pdb3jw0/pdb], 5HPL [https://doi.org/10.2210/pdb5hpl/pdb]. Source data are provided as a Source Data file.

Research involving human participants, their data, or biological material

Policy information about studies with human participants or human data. See also policy information about sex, gender (identity/presentation), and sexual orientation and race, ethnicity and racism.

Reporting on sex and gender	N/A
Reporting on race, ethnicity, or other socially relevant groupings	N/A
Population characteristics	N/A
Recruitment	N/A
Ethics oversight	N/A

Note that full information on the approval of the study protocol must also be provided in the manuscript.

Field-specific reporting

Please select the one below that is the best fit for your research. If you are not sure, read the appropriate sections before making your selection.

[x] Life sciences [ ] Behavioural & social sciences [ ] Ecological, evolutionary & environmental sciences

For a reference copy of the document with all sections, see nature.com/documents/nr-reporting-summary-flat.pdf

Life sciences study design

All studies must disclose on these points even when the disclosure is negative.

Sample size	For Cryo-EM data, images were collected until the resolution of the final 3D reconstruction maps are sufficient for model building. A total of 3,536 movies for E6AP were collected. A total of 21,152 movies for the E6AP/E6 complex were collected. For all functional assay, we use the sample size at least of three independent experiments.
Data exclusions	No data were excluded from the analysis.
Replication	All assays were repeated at least three times, showing similar results.
Randomization	Randomization was not necessary, as the independent variables to be tested were sufficient for the functional interpretation within this study.
Blinding	We were blinded to group allocation during data collection and analysis.

Reporting for specific materials, systems and methods

We require information from authors about some types of materials, experimental systems and methods used in many studies. Here, indicate whether each material, system or method listed is relevant to your study. If you are not sure if a list item applies to your research, read the appropriate section before selecting a response.

Materials & experimental systems

n/a	Involved in the study	
[ ]	[x]	Antibodies
[ ]	[x]	Eukaryotic cell lines
[x]	[ ]	Palaeontology and archaeology
[x]	[ ]	Animals and other organisms
[x]	[ ]	Clinical data
[x]	[ ]	Dual use research of concern
[x]	[ ]	Plants

Methods

n/a	Involved in the study	
[x]	[ ]	ChIP-seq
[x]	[ ]	Flow cytometry
[x]	[ ]	MRI-based neuroimaging

Antibodies

Antibodies used	anti-Flag antibody (ABclonal, AE005), anti-HA antibody (ABclonal, AE008), horseradish peroxidase-conjugated goat anti-mouse antibody (ABclonal, AS003).
Validation	All the antibodies used in the present study are well verified commercial antibodies.<br>anti-Flag antibody: https://abclonal.com.cn/catalog/AE005;<br>anti-HA antibody: https://abclonal.com.cn/catalog/AE008;<br>horseradish peroxidase-conjugated goat anti-mouse antibody: https://abclonal.com.cn/catalog/AS003

Eukaryotic cell lines

Policy information about cell lines and Sex and Gender in Research

Cell line source(s)	Sf9 (Invitrogen, 11496015), Escherichia coli BL21 (DE3) (New England Biolabs).
Authentication	Used as expression stains only, independent verification after purchase not required.
Mycoplasma contamination	Cell lines were tested and free from mycoplasma contamination.
Commonly misidentified lines<br>(See ICLAC register)	No commonly misidentified cell lines were used.

Plants

Seed stocks	N/A
Novel plant genotypes	N/A
Authentication	N/A
